# Association Between Monocyte to High-Density Lipoprotein Cholesterol Ratio and Risk of Non-alcoholic Fatty Liver Disease: A Cross-Sectional Study

**DOI:** 10.3389/fmed.2022.898931

**Published:** 2022-05-19

**Authors:** Liping Wang, Jinzhong Dong, Miao Xu, Li Li, Naibin Yang, Guoqing Qian

**Affiliations:** ^1^Department of Infectious Diseases, Ningbo First Hospital, Ningbo University, Ningbo, China; ^2^Department of Hepatology, Non-alcoholic Fatty Liver Disease (NAFLD) Research Center, Ningbo Hospital of Zhejiang University, Ningbo, China; ^3^Department of Intensive Care Medicine, Ningbo First Hospital, Ningbo University, Ningbo, China; ^4^Department of Endocrinology and Metabolism, Ningbo First Hospital, Ningbo University, Zhejiang, China

**Keywords:** monocyte, NAFLD, high-density lipoprotein cholesterol, vibration controlled and transient elastography, NHANES

## Abstract

**Background:**

Non-alcoholic fatty liver disease (NAFLD) is a global health problem affecting more than a quarter of the entire adult population. Both monocytes and high-density lipoprotein cholesterol (HDL-C) were found to participate in the progression of hepatic inflammation and oxidative stress. We speculated that the monocyte-to-HDL-C ratio (MHR) may be associated with the risk of NAFLD.

**Methods:**

We conducted a cross-sectional study using data from the National Health and Nutrition Examination Survey (NHANES) 2017–2018. NAFLD was identified using a controlled attenuation parameter (CAP) of ≥274 dB/m. Degree of liver fibrosis were assessed by liver stiffness measurement (LSM) and LSM values≥8.0, ≥ 9.7, and ≥13.7 kPa were defined as significant fibrosis (≥F2), advanced fibrosis (≥F3) and cirrhosis (F4), respectively. The association between MHR and the risk of NAFLD and liver fibrosis was estimated using weighted multivariable logistic regression. The non-linear relationship between MHR and the risk of NAFLD was further described using smooth curve fittings and threshold effect analysis.

**Results:**

Of 4,319 participants, a total of 1,703 (39.4%) participants were diagnosed with NAFLD. After complete adjustment for potential confounders, MHR was positively associated with the risk of NAFLD (OR = 2.87, 95% CI: 1.95–4.22). The risk of NAFLD increased progressively as the MHR quarter increased (*P* for trend < 0.001). In subgroup analysis stratified by sex, a positive association existed in both sexes; Women displayed higher risk (men: OR = 2.12, 95% CI: 1.33–3.39; women: OR = 2.64, 95%CI: 1.40–4.97). MHR was positively associated with the risk of significant liver fibrosis (OR = 1.60, 95% CI: 1.08–2.37) and cirrhosis (OR = 1.83, 95% CI: 1.08–3.13), but not with advanced liver fibrosis (OR = 1.53, 95% CI: 0.98–2.39) after full adjustment for potential confounders. In the subgroup analysis by sex, the association between MHR and different degrees of liver fibrosis was significantly positive in women. When analyzing the relationship between MHR and NAFLD risk, a reverse U-shaped curve with an inflection point of 0.36 for MHR was found in women.

**Conclusion:**

Higher MHR was associated with increased odds of NAFLD among Americans of both sexes. However, an association between MHR and liver fibrosis was found mainly among women.

## Introduction

Non-alcoholic fatty liver disease (NAFLD) refers to a fatty liver appearance as well as liver inflammation found in liver biopsy specimens of individuals with metabolic dysfunctions but without significant alcohol consumption (1). NAFLD has become gradually more prevalent over the past four decades and grew to be a global public health concern due to unhealthy lifestyle elements, such as a high-energy diet, sedentary routine, and an absence of exercise. A cross-sectional study estimated that the prevalence of NAFLD was 37.1% among American adults in the general population (2). More than a quarter of the adult population worldwide suffers from NAFLD, and its prevalence is predicted to increase further to 56% in the next decade (3). This disease state is strongly associated with type 2 diabetes mellitus (T2DM), hypertension, chronic kidney disease (4), obesity, and other components of metabolic syndrome (MetS) (5, 6) as well as hepatocellular carcinoma (7, 8); it is responsible for an alarming clinical state, a higher economic burden, and an increased mortality (9). Although higher cardiovascular disease (CVD) risk is observed in patients with NAFLD, an independent association between steatosis, fibrosis, and CVD was not identified in a population-based study (10). On histopathology, NAFLD can be categorized into simple fatty liver, steatohepatitis (NASH), fibrosis, and irreversible cirrhosis; the last disease state tends to eventually degenerate to hepatocellular carcinoma. It was widely proved that NASH patients with fibrosis stage 2 or higher have elevated all-cause and liver-related mortality. Considering liver biopsy has several vital shortcomings, the need for reliable non-invasive tools for the diagnosis, risk stratification and monitoring of the fibrosis course of NAFLD is urgent. Among them the FNIH NIMBLE project is one of the most promising (11). Without the effective treatment recommended by the FDA, a monitor indicator with high sensitivity and specificity for the early detection of NAFLD is urgently required.

The theory of NAFLD pathology proposed by Tilg and Moschen (12), namely the “Multiple-hit hypothesis,” is currently accepted by a majority of researchers. They suggested that multiple simultaneous synergistic events lead to insulin resistance, oxidative stress, and inflammation of the liver. In addition, chronic inflammation facilitates fat accumulation, a precursor state to steatosis in NASH (12, 13). A large amount of free fatty acids (FFAs) reaching the hepatic parenchyma results in alterations in mitochondrial function and increases the production of reactive oxygen species (ROS) (14). It has been shown that ROS increase cannot be effectively counteracted in NAFLD due to inefficiency of the ROS detoxification systems (15) and can also lead to lipid damage by lipid peroxidation. Two recently published articles indicated that oxidative stress in NAFLD is closely related to activation of the immune system and facilitates the activation of Kupffer cells (KCs), which, in turn produce ROS (16, 17). Since inflammation and oxidative stress in the liver are responsible for the progression of NAFLD, markers of inflammation or elements correlated with oxidative stress may act as promising markers of early NAFLD stages. Recently, it was found that a choline-deficient, high-fat diet increases monocyte-derived hepatic macrophages in an *in vitro* model (18). Monocytes are vital innate inflammatory cells responsible for pro-inflammatory cytokine increase (19). Moreover, high-density lipoprotein cholesterol (HDL-C) has been reported to exhibit antioxidant and anti-inflammatory functions in participants with T2DM and atherosclerosis (20). The monocyte-to-HDL-C ratio (MHR) may be a novel marker of inflammation and oxidative stress. Elevated MHR is associated with the occurrence and development of extracranial and intracranial atherosclerotic stenosis (21), non-ST-segment elevation acute coronary syndrome (22), and neuromyelitis optica spectrum disorders (23). However, the relationship between MHR and NAFLD risk has not been fully addressed, especially in the general American population.

We conducted a national representative cross-sectional study based on the 2017–2018 National Health and Nutrition Examination Survey (NHANES) to explore the correlation of MHR with NAFLD risk in the general U.S. population to find a clinically accessible monitoring indicator of NAFLD.

## Methods

### Data Source

To provide objective information on health conditions and identify emerging global public health issues in the US, the National Center for Health Statistics (NCHS) designed and conducted the NHANES, which is an ongoing national representative and cross-sectional survey that collected information on nutrition and health in the US. Data from NHANES can be found at www.cdc.gov/nchs/nhanes/ and is freely and publicly available for researchers throughout the world. The Institutional Review Board of the NCHS ratified the survey protocol and written informed consent was provided by each participant. The NCHS collected participant information, including questionnaires, laboratory tests results, and physical examination findings. In this study, we collected data from the NHANES from 2017–2018, during which 9,254 participants were involved.

**Figure 1:**
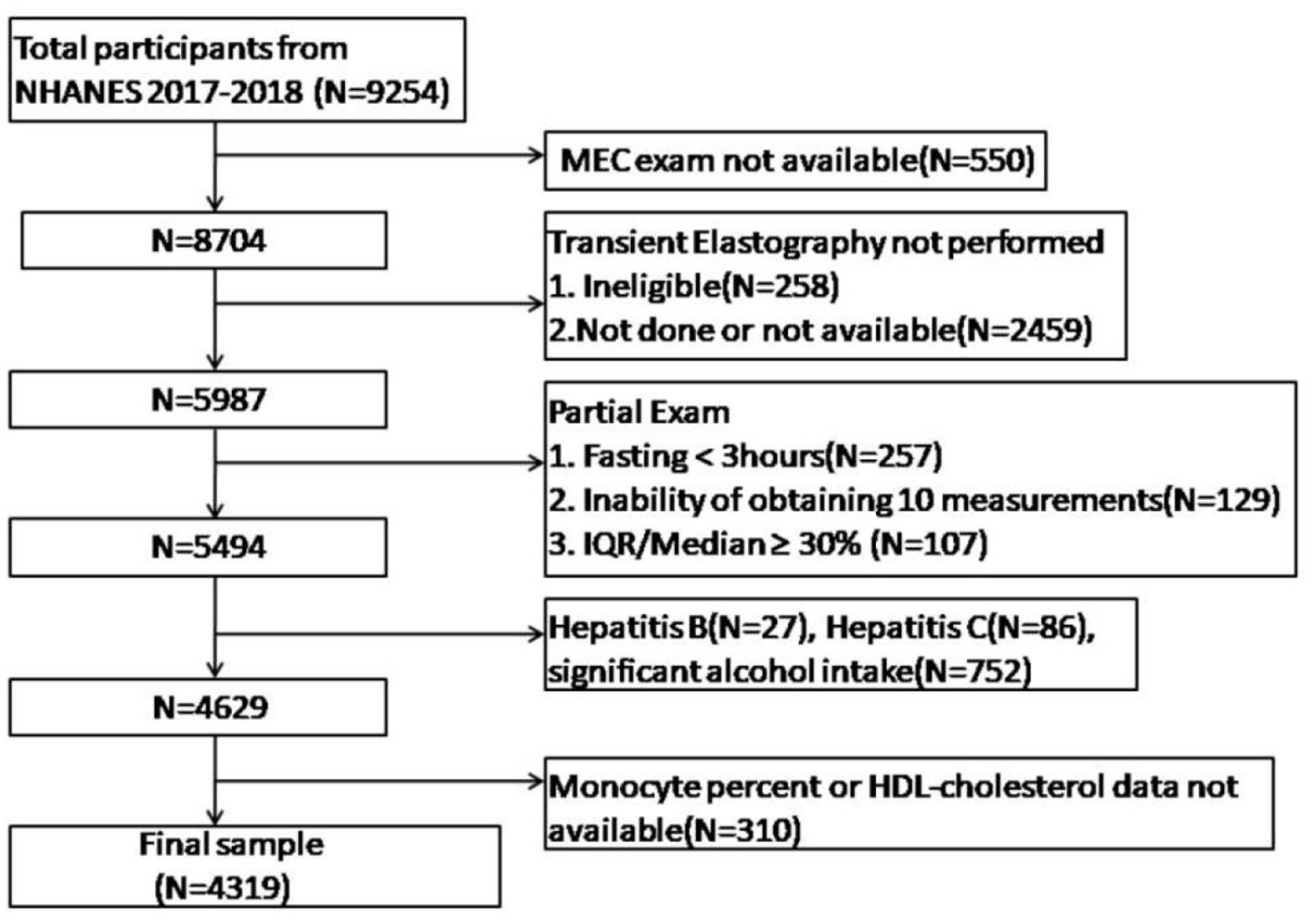
Flowchart of subjects included in this study.

Of these participants, we excluded 550 with missing MEC exams, 2,717 without transient elastography results (including 258 with ineligible transient elastography and 2,459 with not done or unavailable transient elastography), 493 with partial exam [including 257 with fasting <3 h, 129 unable to obtain ten measurements, and 107 with IQR/Median liver stiffness measurement (LSM) values ≥ 30%], 865 with other common liver diseases (including 27 with hepatitis B, 86 with hepatitis C, 752 with significant alcohol intake defined as > 2 standard drinks/day for women and > standard 3 drinks /day for men), and 310 without available monocyte levels or HDL-C data. Eventually, 4,319 participants were included in the current study (Figure 1).

### Vibration Controlled and Transient Elastography

Liver biopsy remains the gold standard for the assessment of hepatic steatosis and fibrosis. However, this procedure has been reported to have possible complications, including bleeding or even death (1: 10,000) (24), is costly, and has poor reproducibility. It is currently being gradually replaced by vibration-controlled and transient elastography (VCTE) in clinical practice. VCTE is a rapid, non-invasive imaging modality validated for assessing the degree of liver fibrosis and steatosis, with acceptable accuracy. In VCTE, controlled attenuation parameter (CAP) are used with a cutoff of ≥274 dB/m for steatosis (25). Liver stiffness measurement (LSM) values of ≥8.0, ≥9.7, and ≥13.7 kPa correspond to significant fibrosis (≥F2), advanced fibrosis (≥F3) and cirrhosis (F4), respectively (26). To avoid possible bias, cut-off selection was confirmed before data analysis in our study.

### Variables

MHR was considered as the independent variable, and the prevalence of NAFLD and liver fibrosis was regarded as the dependent variable. All participants fasted for more than 3 h before undergoing laboratory tests. Measurements of liver enzymes, glucose, uric acid, serum lipids, and monocyte counts were obtained using a venous blood sample auto analyzer. MHR was calculated as the ratio of monocyte counts (10^9^ cells /L) to HDL-C level (mmol/L). NAFLD was diagnosed according to the following criteria: (i) CAP values ≥274 dB/m detected using FibroScan. (ii) Exclusion of other etiologies of chronic liver disease (significant alcohol intake, defined as > 2 standard drinks/day for women and >3 standard drinks /day for men; b. Hepatitis B or C virus infection).

### Covariates

Demographic parameters such as age, sex, and race/ethnicity were obtained through self-reported questionnaires. Other covariates, including weight, height, waist circumference, systolic blood pressure (SBP), and diastolic blood pressure (DBP) were measured by the NHANES staff. Body mass index (BMI) was calculated by dividing the height (cm) by the weight (kg) in meters squared. T2DM was diagnosed according to the following criteria (27): (1) self-reported diabetes; (2) use of antidiabetic medicines; (3) fasting plasma glucose (FPG) ≥126 mg/dl (7 mmol/L); (4) 2-h plasma glucose ≥ 200 mg/dl (11.1 mmol/L) during an oral glucose tolerance test (OGTT); (5) hemoglobin A1c ≥ 6.5% (48 mmol/mol); and (6) with classic symptoms of hyperglycemia or hyperglycemic crisis, random plasma glucose level ≥ 200 mg/dL (11.1 mmol/L). A standard biochemical profile, including total cholesterol, triglyceride, glycohemoglobin, low-density lipoprotein cholesterol (LDL-C), aspartate aminotransferase (AST), alanine aminotransferase (ALT), γ-glutamyltranspeptidase (GGT), serum albumin, uric acid, platelet count, fasting glucose, and serum creatinine was also performed. The covariate acquisition process is available at https://www.cdc.gov/nchs/nhanes/.

### Statistical Methods

To ensure national representation, weighted analyses were performed according to the guidelines recommended by the NCHS. Continuous variables were described as a weighted mean and weighted standard deviation if normally distributed, or otherwise as median and interquartile range (IQR). Categorical variables were described as frequencies and weighted proportions. Package R version 3.4.3 (http://www.R-project.org) and EmpowerStats software were used to analyze the data. Student's *t*-test, Mann-Whitney *U*-test, and chi-squared (χ2) tests were performed to compare anthropometric and laboratory parameters when needed. The statistical significance level was set at *P* < 0.05 (two-tailed). Unadjusted and adjusted logistic regression models were created to investigate the association between MHR and NAFLD and liver fibrosis. Model 1: no covariates were adjusted for; Model 2: age, sex, and race were adjusted for; Model 3: age, sex, race, BMI, hypertension, DM, smoking, ALT, total cholesterol, PLT, albumin, and statin use were adjusted for. After a quarter classification of MHR, weighted multivariable logistic regression analysis and *P* for trend were calculated. A subgroup analysis was performed after stratification by sex. Smooth curve fittings and threshold effect analysis were used to assess the potential non-linear relationship.

## Results

A total of 4,319 participants were included in the study. Among these, 1,703 (39.4%) were diagnosed with NAFLD. In contrast with participants without NAFLD, patients with NAFLD were more likely to be older, men, non-Hispanic white, with hypertension, using statins, and of higher BMI, blood pressure, total cholesterol, triglyceride, glycohemoglobin, LDL-C, liver enzymes, uric acid, fast glucose, and serum creatinine levels; patients with NAFLD were also more likely to have lower serum albumin levels (*P* < 0.05). Moreover, MHR in patients with NAFLD was higher than that in the non-NAFLD group (0.49 ± 0.25 vs. 0.39 ± 0.16, *P* < 0.0001). The results are presented in Table 1.

**Table 1:** Weighted characteristic of the participants according to NAFLD.

**Parameters**	**Total**	**Non-NAFLD**	**NAFLD group**	***P*-value**
	**(*N* = 4,319)**	**(*N* = 2,616)**	**(*N* = 1,703)**	
Age (years)	44.53 ± 19.81	40.46 ± 19.98	51.17 ± 17.63	<0.0001
Sex: *n* (%)				0.0006
Men	49.03	46.11	53.79	
Women	50.97	53.89	46.21	
Race/Ethnicity: *n* (%)				0.0015
Mexican American	9.30	7.85	11.66	
Other Hispanic	7.14	7.91	5.88	
Non-Hispanic White	60.64	59.57	62.38	
Non-Hispanic Black	11.70	12.70	10.08	
Non-Hispanic Asian	6.38	6.28	6.56	
Other race	4.84	5.69	3.45	
Diabetes status				0.3098
Yes	10.14	9.7	10.85	
No	89.86	91.3	89.15	
Hypertension: *n* (%)	1,026 (23.76%)	489 (18.69%)	537 (31.53%)	<0.001
Smoking: *n* (%)				<0.001
Current smoker	469 (10.85%)	293 (11.20%)	176 (10.33%)	
Former smoker	870 (20.13%)	431 (16.47%)	439 (25.76%)	
Non-smoker	2,980 (69.01%)	1,892 (72.33%)	1,088 (63.91%)	
Statin use: *n* (%)	818 (18.93%)	352 (13.45%)	466 (27.35%)	<0.001
BMI (Kg/m^2^)	28.63 ± 6.95	26.02 ± 5.64	32.90 ± 6.76	<0.0001
Waist circumference (cm)	97.49 ± 17.11	90.51 ± 14.38	108.88 ± 14.95	<0.0001
SBP	132.97 ± 6.71	132.74 ± 6.67	133.32 ± 6.75	0.018
DBP	75.97 ± 4.57	75.89 ± 4.00	76.10 ± 5.34	<0.001
**Laboratory features**
Total cholesterol (mg/dL)	183.48 ± 41.51	180.74 ± 40.36	187.95 ± 42.82	0.0001
Triglyceride (mg/dL)	107.78 ± 98.41	87.23 ± 55.80	141.31 ± 136.33	<0.0001
Glycohemoglobin (%)	5.64 ± 0.89	5.44 ± 0.62	5.96 ± 1.12	<0.0001
LDL-C	2.77 ± 0.64	2.74 ± 0.63	2.82 ± 0.65	<0.001
AST (IU/L)	21.00 ± 9.91	20.42 ± 10.27	21.94 ± 9.22	0.0008
ALT (IU/L)	21.19 ± 15.29	18.63 ± 14.14	25.38 ± 16.15	<0.0001
GGT (IU/L)	25.61 ± 27.98	21.61 ± 23.42	32.15 ± 33.11	<0.0001
Serum albumin (g/L)	40.76 ± 3.18	41.20 ± 3.16	40.05 ± 3.08	<0.0001
Uric acid (μmol/L)	320.01 ± 83.71	304.06 ± 79.54	346.03 ± 83.81	<0.0001
PLT (10^9^/L)	240.95 ± 61.17	239.85 ± 60.60	242.73 ± 62.05	0.3027
Fast glucose (mmol/L)	6.07 ± 1.68	5.69 ± 1.05	6.68 ± 2.23	<0.0001
Serum creatinine (μmol/L)	75.73 ± 25.48	74.49 ± 25.16	77.76 ± 25.87	0.0049
MHR	0.43 ± 0.21	0.39 ± 0.16	0.49 ± 0.25	<0.0001

*Mean ± SD was for continuous variables. P-value was calculated by weighted linear regression model. % was for categorical variables. P-value was calculated by weighted chi-square test*.

The associations between MHR and the prevalence of NAFLD were positive in all multivariable logistic regression models (model 1: OR = 10.86, 95% CI: 7.90–14.94; model 2: OR = 10.56, 95% CI: 7.48–14.92; model 3: OR = 2.87, 95% CI: 1.95–4.22). Notably, after fully adjusting for potential confounders, every one-unit increase in MHR was associated with a 1.87-fold NAFLD risk increase. Moreover, the risk of NAFLD increased more progressively in the higher quartile groups of MHR when compared to the lowest quartile group (*P* for trend < 0.001). The results are presented in Table 2.

**Table 2:** The correlation between MHR and risk of NAFLD.

**Model**	**Model 1: OR (95% CI) *P*-value**	**Model 2: OR (95% CI) *P*-value**	**Model 3: OR (95% CI) *P*-value**
MHR	10.86 (7.90, 14.94) <0.0001	10.56 (7.48, 14.92) <0.0001	2.87 (1.95, 4.22) <0.0001
**MHR (Quartile)**
Q1 (0.04–0.30)	Reference	Reference	Reference
Q2 (0.30–0.41)	1.68 (1.39, 2.03) <0.0001	1.73 (1.42, 2.10) <0.0001	1.34 (1.07, 1.68) 0.0104
Q3 (0.41–0.55)	2.46 (2.05, 2.96) <0.0001	2.49 (2.05, 3.03) <0.0001	1.61 (1.28, 2.02) <0.0001
Q4 (0.55–6.15)	3.88 (3.23, 4.66) <0.0001	3.85 (3.16, 4.70) <0.0001	1.92 (1.51, 2.43) <0.0001
*P* for trend	<0.001	<0.001	<0.001
**Subgroup analysis stratified by sex**
Men	11.00 (7.14, 16.94) <0.0001	9.52 (6.08, 14.88) <0.0001	2.12 (1.33, 3.39) 0.0017
Women	9.14 (5.48, 15.25) <0.0001	11.76 (6.82, 20.29) <0.0001	2.64 (1.40, 4.97) 0.0027

*Model 1: No covariates were adjusted. Model 2: Age, sex, and race were adjusted. Model 3: age, sex, race, BMI, hypertension; DM, smoking; ALT, total cholesterol; PLT, albumin and statin use were adjusted. In the subgroup analysis, sex was not adjusted*.

A subgroup analysis stratified by sex was performed. In men, the positive associations were statistically significant in all models (model 1: OR = 11.00, 95% CI: 7.14–16.94; model 2: OR = 9.52, 95% CI: 6.08–14.88; model 3: OR = 2.12, 95% CI: 1.33–3.39). In women, we also found a positive association in each model (model 1: OR = 9.14, 95% CI: 5.48–15.25; model 2: OR = 11.76, 95% CI: 6.82–20.29; model 3: OR = 2.64, 95% CI: 1.40–4.97).

In the fully adjusted multivariable logistic regression models, the associations between MHR and the prevalence of significant liver fibrosis (OR = 1.60, 95% CI = 1.08–2.37) and liver cirrhosis (OR= 1.83, 95% CI = 1.08–3.13) were significantly positive, but not between MHR and the prevalence of advanced liver fibrosis (OR = 1.53, 95% CI: 0.98–2.39). After subgroup analysis by sex, associations between MHR and the prevalence of liver fibrosis persisted and were more significant in women (significant liver fibrosis: OR = 4.71, 95% CI: 1.84–12.07; advanced liver fibrosis: OR = 6.10, 95% CI: 1.91–19.46; liver cirrhosis: OR = 13.02, 95% CI: 2.49–68.11). However, no significant associations were found in men (significant liver fibrosis: OR = 1.23, 95% CI: 0.77–1.94; advanced liver fibrosis: OR = 1.06, 95% CI: 0.56–1.99; liver cirrhosis: OR = 13.02, 95% CI: 2.49–68.11). The results are presented in Table 3.

**Table 3:** The association between MHR and risk of liver fibrosis.

**Degree of liver fibrosis**		**Model 1**	**Model 2**	**Model 3**
		**OR (95% CI) *P*-value**	**OR (95% CI) *P*-value**	**OR (95% CI) *P*-value**
Significant fibrosis (F2)	MHR	3.51 (2.40, 5.13) <0.0001	3.11 (2.08, 4.65) <0.0001	1.60 (1.08, 2.37) 0.0182
(LSM ≥ 8.0)	Men	2.30 (1.50, 3.51) 0.0001	2.20 (1.41, 3.43) 0.0005	1.23 (0.77, 1.94) 0.3859
	Women	8.88 (4.07, 19.38) <0.0001	8.86 (3.98, 19.75) <0.0001	4.71 (1.84, 12.07) 0.0013
Advanced fibrosis (F3)	MHR	3.06 (1.99, 4.70) <0.0001	2.51 (1.59, 3.97) <0.0001	1.53 (0.98, 2.39) 0.0586
(LSM ≥ 9.7)	Men	2.03 (1.30, 3.19) 0.0020	1.85 (1.16, 2.95) 0.0101	1.06 (0.56, 1.99) 0.8636
	Women	9.94 (3.77, 26.16) <0.0001	8.98 (3.34, 24.11) <0.0001	6.10 (1.91, 19.46) 0.0023
Cirrhosis (F4)	MHR	2.77 (1.63, 4.68) 0.0002	2.24 (1.31, 3.84) 0.0033	1.83 (1.08, 3.13) 0.0260
(LSM ≥ 13.7)	Men	2.07 (1.23, 3.49) 0.0063	1.80 (1.06, 3.05) 0.0284	1.25 (0.52, 2.96) 0.6188
	Women	13.93 (3.46, 56.05) 0.0002	10.54 (2.60, 42.68) 0.0010	13.02 (2.49, 68.11) 0.0024

*Model 1: No covariates were adjusted. Model 2: Age, sex, and race were adjusted. Model 3: age, sex, race, BMI, hypertension; DM, smoking; ALT, total cholesterol; PLT, albumin and statin use were adjusted. In the subgroup analysis, sex was not adjusted*.

**Figure 2:**
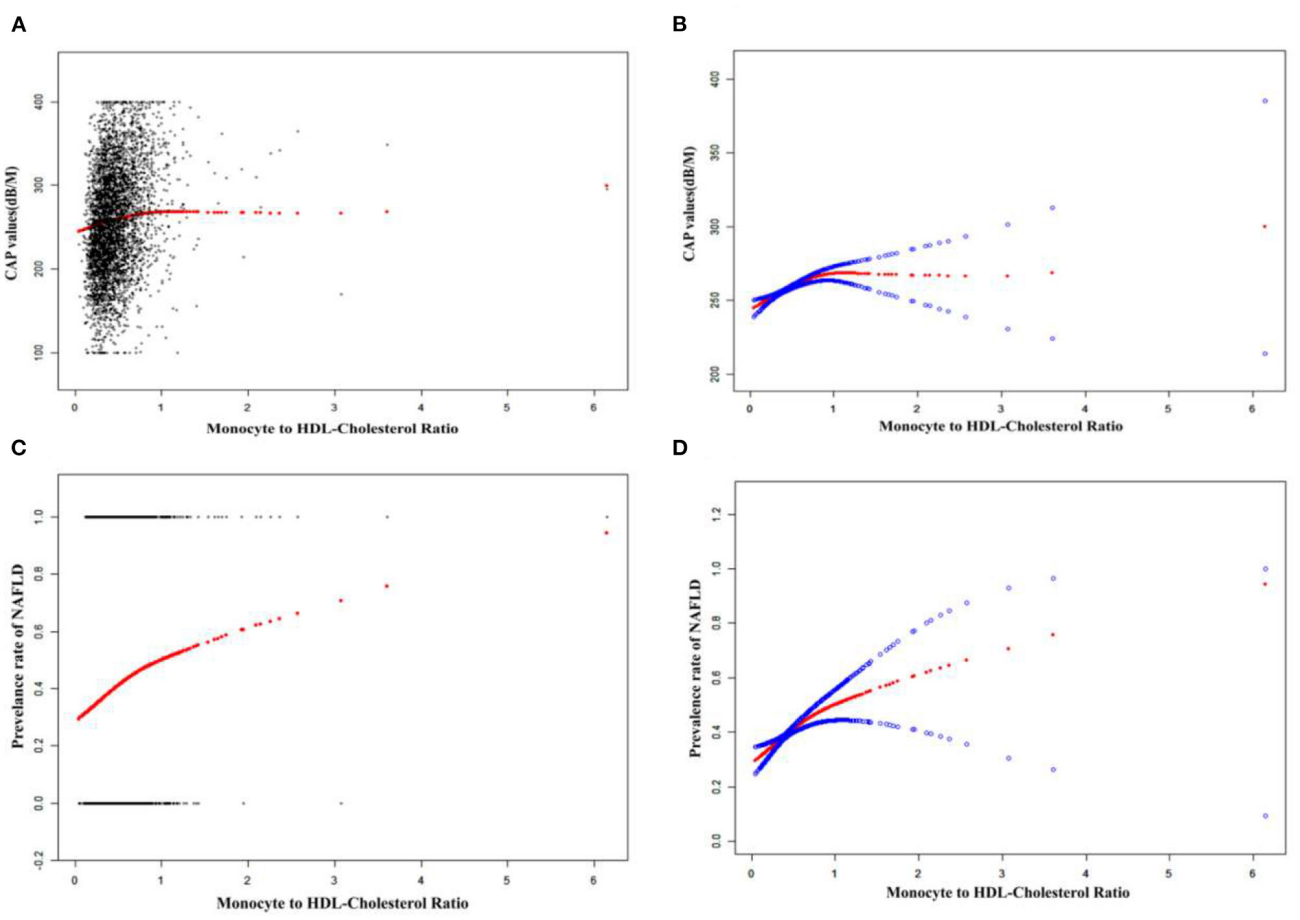
The associations between MHR and CAP values or the prevalence of NAFLD. **(A,C)**: Each black point represents a sample. **(B,D)**: Solid redline represents the smooth curve fit between variables. Blue bands represent the 95% of confidence interval from the fit. Adjust for: age, sex, race, BMI, hypertension; DM, smoking; ALT, total cholesterol; PLT, albumin and statin use.

**Figure 3:**
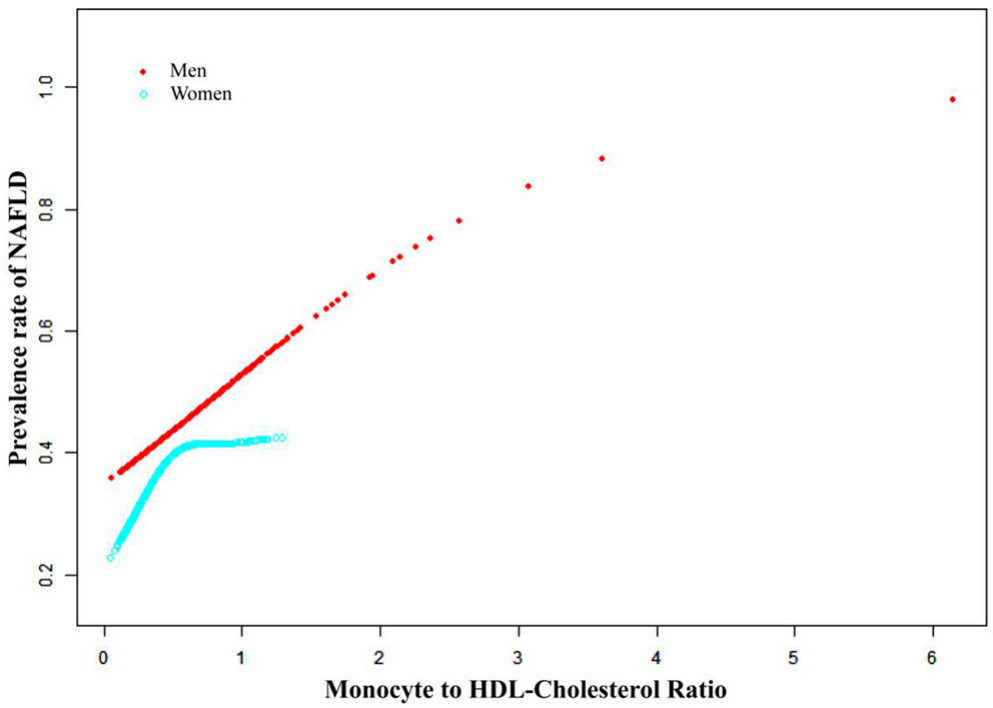
The association between MHR and the prevalence of NAFLD by sex. Adjust for: age, race, BMI, hypertension; DM, smoking; ALT, total cholesterol; PLT, albumin and statin use.

Smooth curve fitting and threshold effect analysis were used to detect the potential non-linear relationship between MHR and the risk of NAFLD. A reverse U-shaped curve, with an inflection point of 0.36, was found among women as shown in Table 4 and Figures 2, 3.

**Table 4:** Threshold effect analysis of MHR on the prevalence of NAFLD in different sexes using the two-piecewise linear regression model.

**Parameters**	**Adjusted OR (95% CI), *P*-value**
**All participants**
Fitting by the standard linear model	2.87 (1.95, 4.22) <0.0001
**Fitting by the two-piecewise linear model**
Inflection point	0.55
MHR <0.55	6.94 (3.39, 14.20) <0.0001
MHR > 0.55	1.54 (0.94, 2.53) 0.0858
Log likelihood ratio	0.004
**Women**
Fitting by the standard linear model	3.90 (2.07, 7.35) <0.0001
**Fitting by the two-piecewise linear model**
Inflection point	0.36
MHR <0.36	74.68 (10.41, 535.81) <0.0001
MHR > 0.36	1.56 (0.67, 3.64) 0.3039
Log likelihood ratio	0.002

*Age, sex, race, BMI, hypertension; DM, smoking; ALT, total cholesterol; PLT, albumin and statin use were adjusted. In the subgroup analysis, sex was not adjusted*.

## Discussion

NAFLD is a common disease that every practicing physician, both hepatologist and non-specialist, would encounter. It is important to aware how to diagnosis, what are the clinical features and complications, and when to refer (28). Especially, an effective monitor indicator for the early detection of NAFLD is urgently required. In the present cross-sectional study, we describe a positive and independent correlation between MHR and the risk of NAFLD and liver fibrosis in a sample US population. Notably, after fully adjusting for potential confounders, every one-unit increase in MHR was associated with a 1.87-fold NAFLD risk increase. Statistically significant positive associations persisted in both sexes. We further revealed positive associations between MHR and the prevalence of significant liver fibrosis and cirrhosis. However, the positive associations between MHR and significant liver fibrosis, advanced liver fibrosis, or liver cirrhosis were more significant in women than in men. Moreover, a reverse U-shaped curve with an inflection point of 0.36 was found among women. These findings indicate that MHR has the potential to be used as a monitoring indicator of NAFLD, and might facilitate the early detection of NAFLD.

Only a few studies have focused on the association between MHR and NAFLD, and the outcomes remain controversial. A recently reported retrospective study involving 409 patients showed that there was a significant positive association between MHR and age, ALT and HOMA-IR values, and the risk of non-alcoholic hepatic steatosis (29). Huang et al. investigated the association between MHR and NAFLD in a Chinese population of 14,189 participants and revealed that MHR was positively associated with the risk of NAFLD diagnosed by hepatic ultrasonography after multivariate logistic regression analysis (30). Our study draws a similar conclusion. Here, we reconfirmed the positive association in a large-scale general American population, and NAFLD was diagnosed using FibroScan, a widely used non-invasive technique to assess the prevalence and severity of NAFLD. In the subgroup analysis stratified by sex, the positive association between the two groups was also statistically significant; women displayed a stronger association between MHR and NAFLD. A U-shaped curve with an inflection point of 0.36 was found among women when analyzing the relationship between MHR and the prevalence of NAFLD. Therefore, in predicting the risk of NAFLD, we suggest evaluating the role of MHR in different sexes.

Hepatocyte injury, caused in the progress of NAFLD by metabolic dysfunctions, releases warning signals related to immune cell recruitment and activation. Hepatic infiltration macrophages, which constitute the largest proportion of resident macrophages in the human body, consist of different cell populations, including monocyte-derived macrophages and resident macrophages named Kupffer cells, which play an important role in NAFLD progression and liver fibrosis (31). Some recent reports have indicated that there are distinct hepatic macrophages in the liver, with M1 macrophages having pro-inflammatory and antimicrobial activity, and an M2 phenotype with anti-inflammatory and reparative functions (32, 33). Different macrophages are involved in the regulation of both hepatic inflammation and homeostasis. As the M1 phenotype is more frequently expressed, after activation, infiltrating hepatic macrophages can facilitate inflammation and liver cirrhosis by releasing pro-inflammatory interleukin 1β and TNF-α, and produce transforming growth factor-β and platelet-derived growth factor (34–36). Furthermore, the soluble macrophage activation marker CD163 has been shown to be associated with liver injury and is a promising predictor for advanced fibrosis (37). In the pathogenesis of NAFLD, after secondary stimulation with an endogenous or exogenous insult, monocytes undergo epigenetic changes (38, 39)and stimulate innate immunity. In addition, HDL-C was found to restrain the production of oxidized low-density lipoprotein cholesterol and suppress the proliferation of monocytes, resulting in antioxidative and anti-inflammatory effects, which are assumed to be responsible for the pathogenesis of NAFLD (40). As both monocytes and HDL-C play important roles in the progression of NAFLD, several studies have reported that MHR could serve as a novel, cost-effective predictor of inflammation, especially in cardiovascular events (41) and metabolic disorders (42). Besides, the presence of fibrosis may affect lipid production in the liver and hepatic fibrosis can distort the hepatic articulture of the hepatocytes causing an altered production of lipids (43). This might partly explain the positive associations between MHR and the prevalence of significant liver fibrosis and cirrhosis found in our current study. However, further studies focusing on the exact mechanism of MHR in NAFLD and exploring the difference between sexes are needed, be it using patient fatty liver biopsy samples or in animal and cell models.

One strength of our study is the large number of participants. However, this study has several limitations. First, the intrinsic mechanism of the relationship between MHR and NAFLD was not elucidated because this was a cross-sectional study. Second, hepatic steatosis in the definition of NAFLD was determined by CAP values, but not by liver biopsy. Third, several therapeutic drugs for dyslipidemia, hypertension, and diabetes mellitus have been shown to affect monocyte counts and HDL-C levels, and could serve as potential confounders. These issues should be addressed in future studies.

## Conclusion

In conclusion, a higher MHR was associated with increased risk of NAFLD among Americans of both sexes. However, an association between MHR and liver fibrosis was found mainly among women. MHR could be used a potential predictor of NAFLD and NAFLD-related liver fibrosis.

## Data Availability Statement

The original contributions presented in the study are included in the article/supplementary material, further inquiries can be directed to the corresponding author/s.

## Ethics Statement

The ethics review board of the National Center for Health Statistics approved all NHANES protocols. The patients/participants provided their written informed consent to participate in this study.

## Author Contributions

NY, GQ, and LL contributed to the conception and study design. NY and MX were in charge of execution, acquisition of data, analysis, and interpretation. LW and JD took charge of drafting, revising, and critically reviewing the article. All authors gave final approval of the version to be published, had agreed on the journal of which the article has been submitted and agreed to be accountable for all aspects of the work.

## Funding

This research was supported by the First Batch of Young Technical Backbone Talents Project of Ningbo Municipal Health Commission (to NY), TianQing Liver Diseases Research Fund Subject of Chinese Foundation for Hepatitis Prevention and Control (No: TQGB20180358), and Major Program of Social Development of Ningbo Science and Technology Bureau (Grant No: 2019C50094).

## Conflict of Interest

The authors declare that the research was conducted in the absence of any commercial or financial relationships that could be construed as a potential conflict of interest.

## Publisher's Note

All claims expressed in this article are solely those of the authors and do not necessarily represent those of their affiliated organizations, or those of the publisher, the editors and the reviewers. Any product that may be evaluated in this article, or claim that may be made by its manufacturer, is not guaranteed or endorsed by the publisher.

## Abbreviations

NHANES: national health and nutrition examination survey
MHR: monocyte counts to HDL-C
HDL-C: high-density lipoprotein cholesterol
LDL-C: low-density lipoprotein cholesterol
VCTE: vibration controlled and transient elastography
BMI: body mass index
AST: aspartate aminotransferase
ALT: alanine aminotransferase
GGT: gamma glutamyl transpeptidase
CAP: controlled attenuation parameter
LSM: liver stiffness measurement
UA: uric acid
DBP: diastolic blood pressure
SBP: systolic blood pressure
DBP: diastolic blood pressure.
